# Supplementary material for: SUMO-targeted Ubiquitin Ligases as crucial mediators of protein homeostasis in Candida glabrata
Source: PLoS Pathog. 2024 Dec 6;20(12):e1012742. doi: 10.1371/journal.ppat.1012742 (PMC11654969; doi:10.1371/journal.ppat.1012742)
Supplement: S1 Fig — Maps of proteins with their domains were produced using IBS 1.0. (PDF) [file ppat.1012742.s001.pdf]

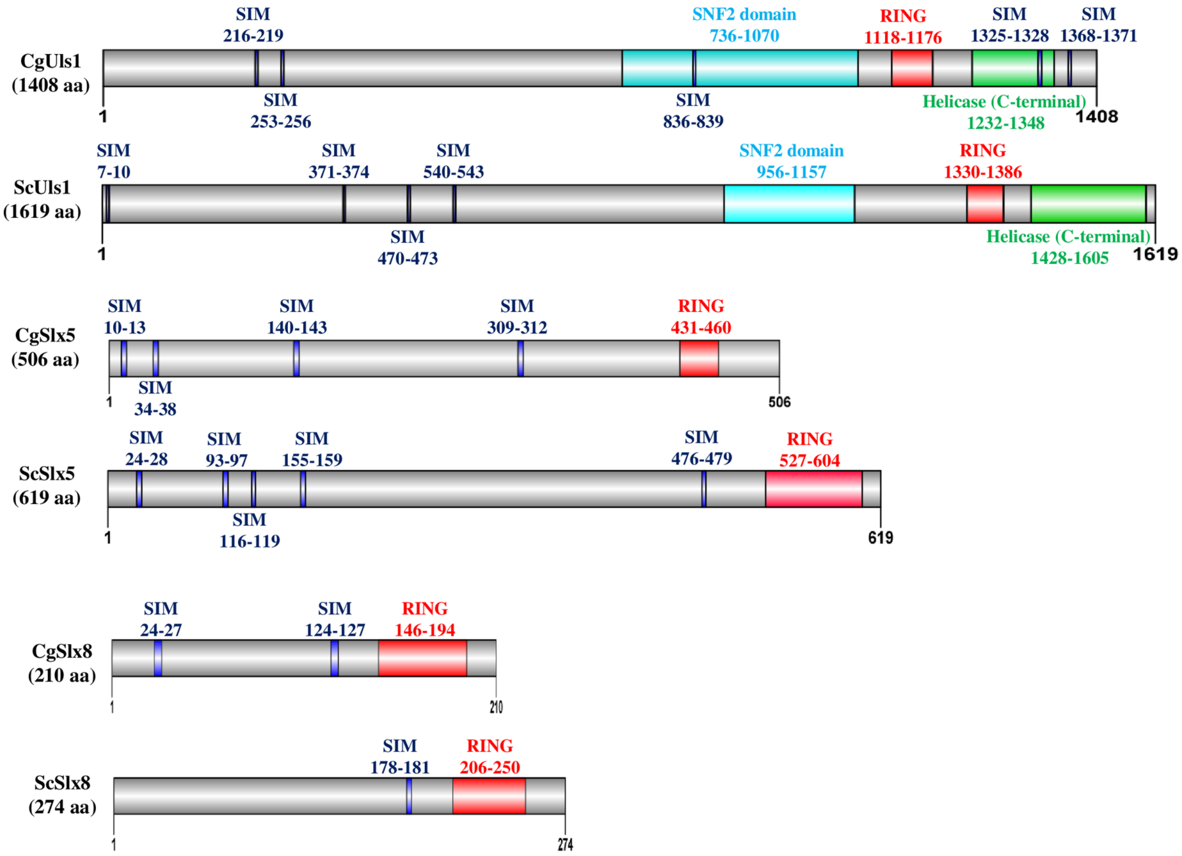

S1 Fig. Schematic representation of orthologs of the *S. cerevisiae* STUbL proteins Slx5, Slx8, Uls1 in *Candida glabrata.* Maps of proteins with their domains were produced using IBS 1.0.
