# Supplementary figures and images for: SUMO-targeted Ubiquitin Ligases as crucial mediators of protein homeostasis in Candida glabrata
Source: PLoS Pathog. 2024 Dec 6;20(12):e1012742. doi: 10.1371/journal.ppat.1012742 (PMC11654969; doi:10.1371/journal.ppat.1012742)

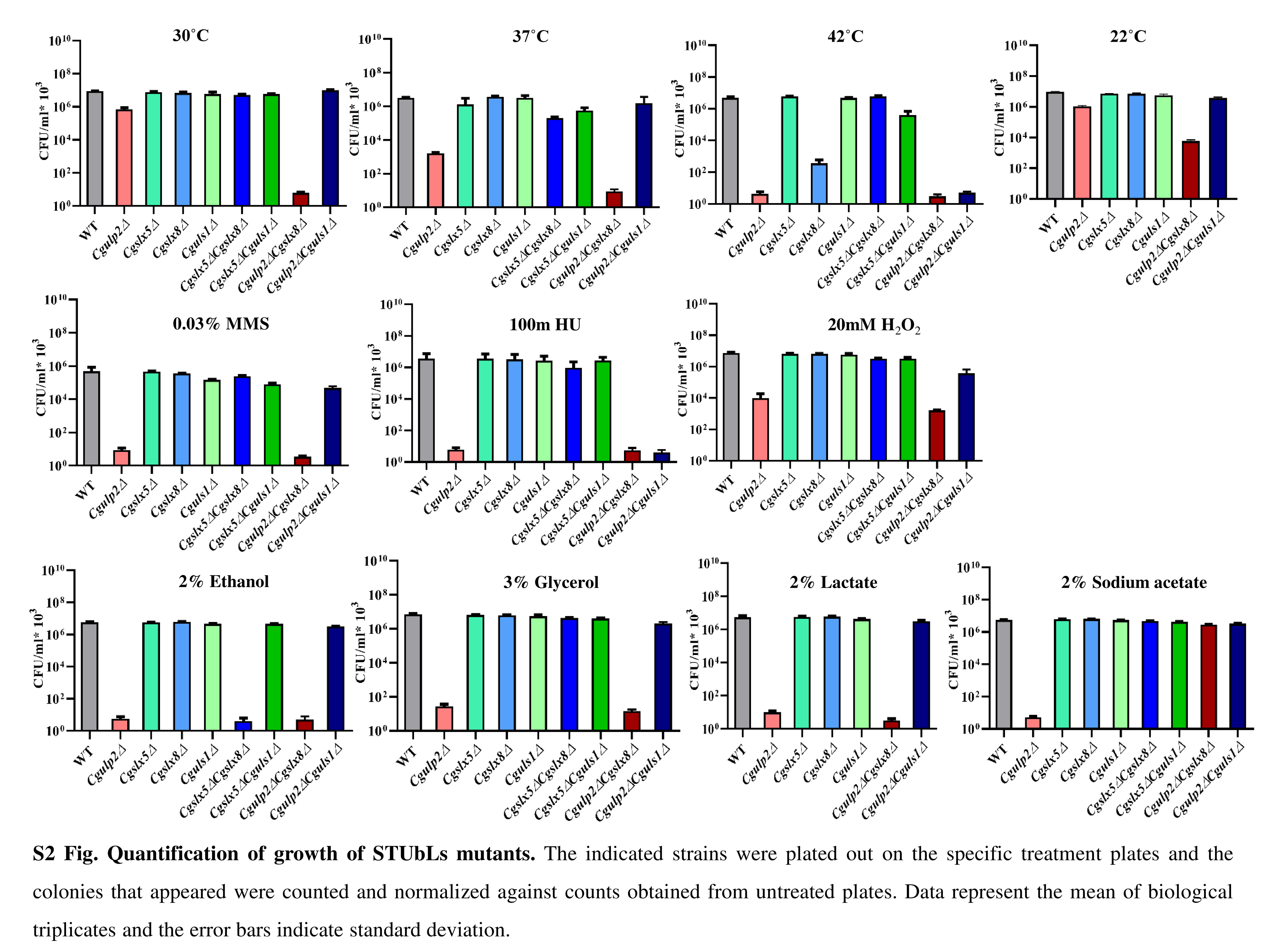

Supplement: S2 Fig — The indicated strains were plated out on the specific treatment plates and the colonies that appeared were counted and normalized against counts obtained from untreated plates. Data represent the mean of biological triplicates and the error bars indicate standard deviation. (TIF) [file ppat.1012742.s002.tif]

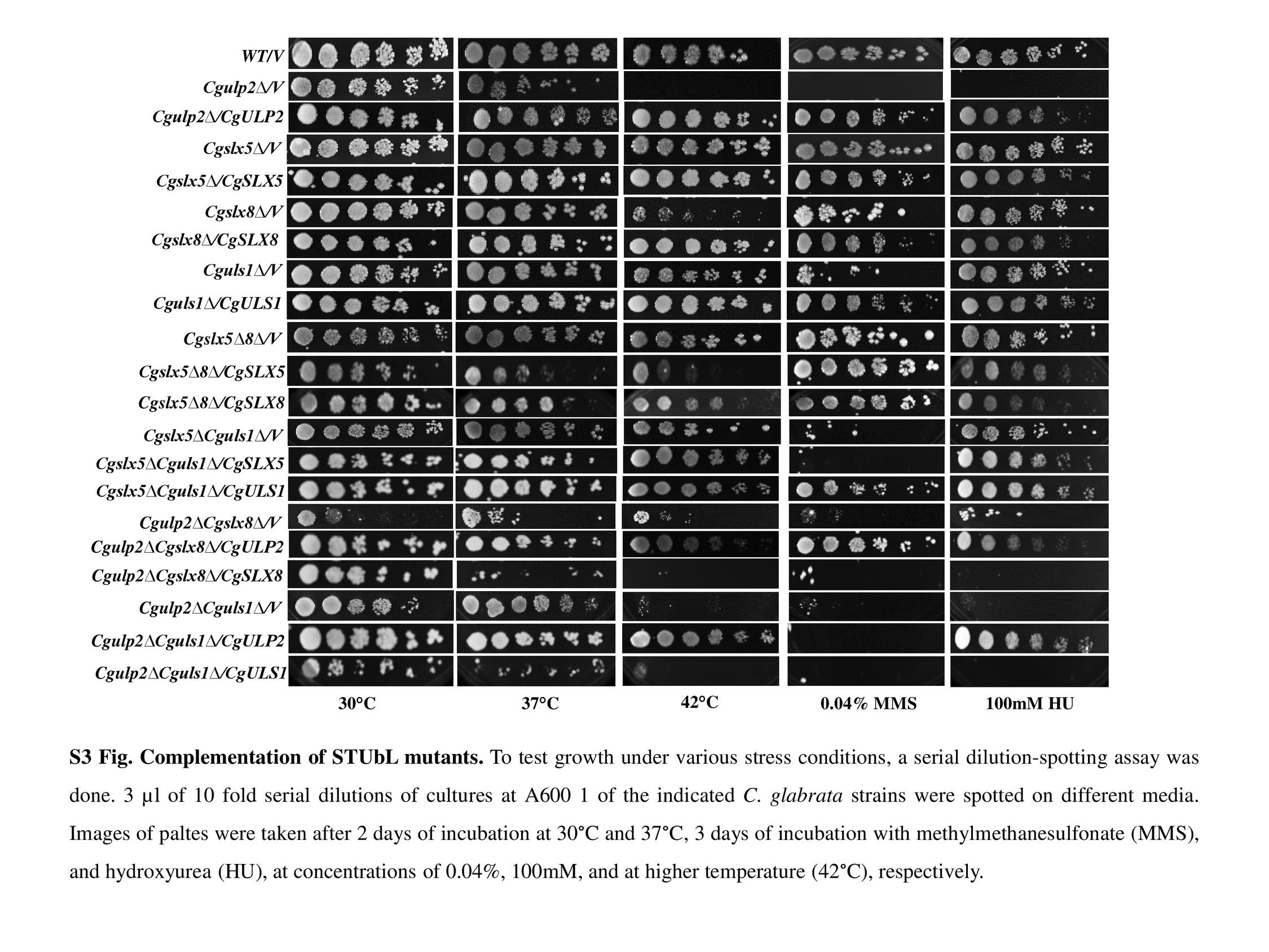

Supplement: S3 Fig — To test growth under various stress conditions, a serial dilution-spotting assay was done. 3 μl of 10 fold serial dilutions of cultures at A600 1 of the indicated C. glabrata strains were spotted on different media. Images of paltes were taken after 2 days of incubation at 30°C and 37°C, 3 days of incubation with methylmethanesulfonate (MMS), and hydroxyurea (HU), at concentrations of 0.04%, 100mM, and at higher temperature (42°C), respectively. (TIF) [file ppat.1012742.s003.tif]

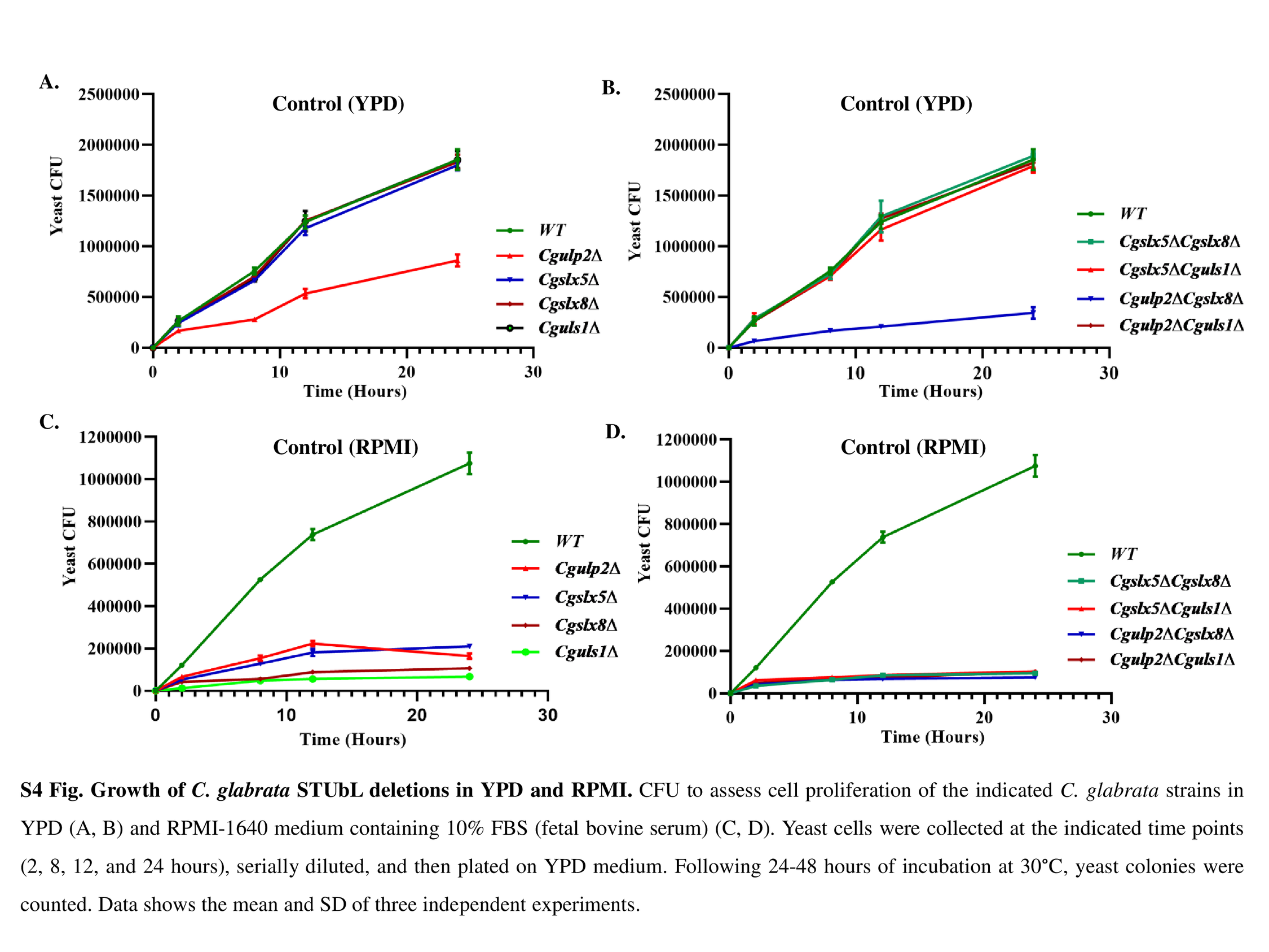

Supplement: S4 Fig — CFU to assess cell proliferation of the indicated C. glabrata strains in YPD (A, B) and RPMI-1640 medium containing 10% FBS (fetal bovine serum) (C, D). Yeast cells were collected at the indicated time points (2, 8, 12, and 24 hours), serially diluted, and then plated on YPD medium. Following 24–48 hours of incubation at 30°C, yeast colonies were counted. Data shows the mean and SD of three independent experiments. (TIF) [file ppat.1012742.s004.tif]

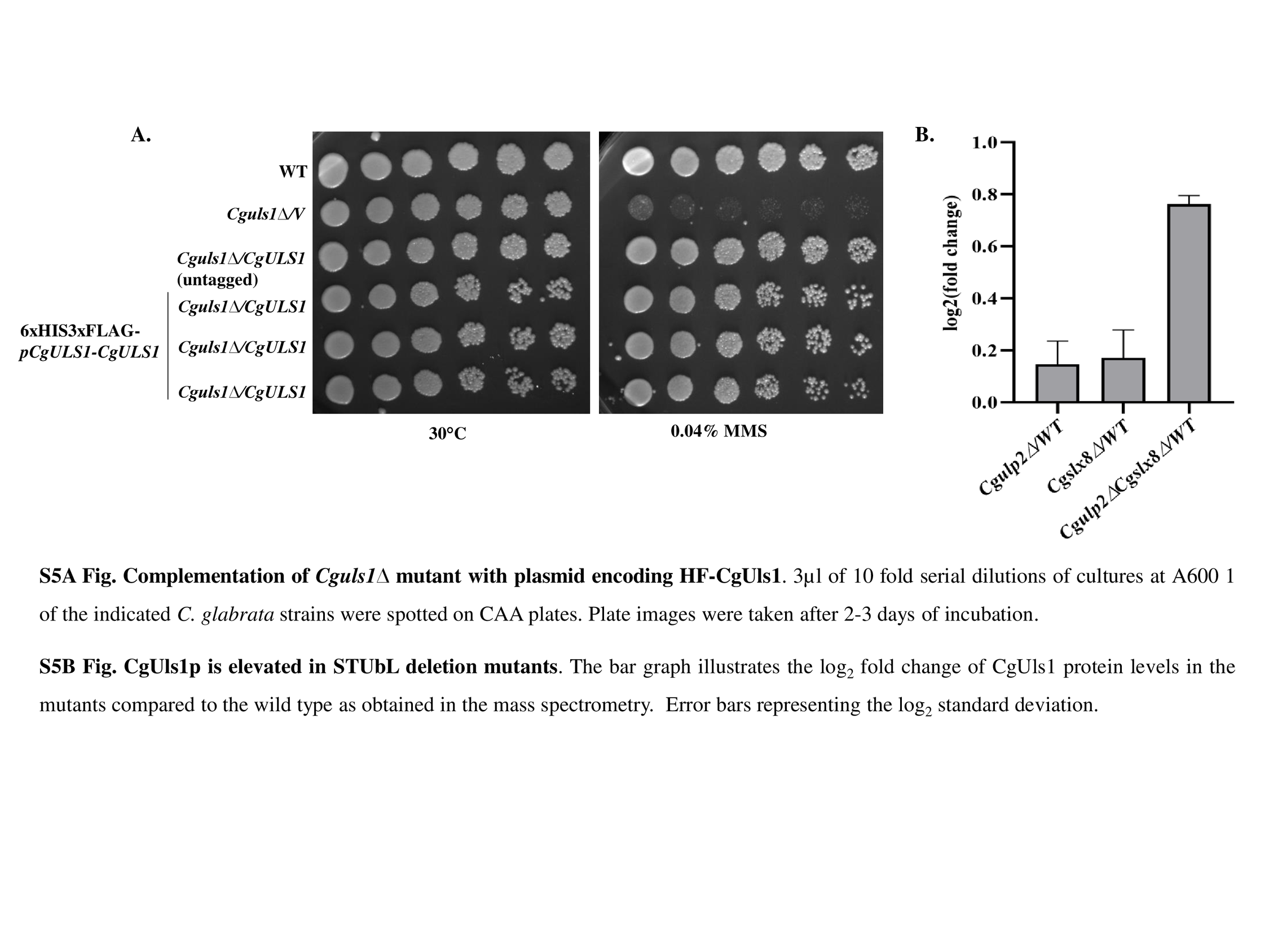

Supplement: S5 Fig — 3μl of 10 fold serial dilutions of cultures at A600 1 of the indicated C. glabrata strains were spotted on CAA plates. Plate images were taken after 2–3 days of incubation. B. CgUls1p is elevated in STUbL deletion mutants. The bar graph illustrates the log2 fold change of CgUls1 protein levels in the mutants compared to the wild type as obtained in the mass spectrometry. Error bars representing the log2 standard deviation. (TIF) [file ppat.1012742.s005.tif]

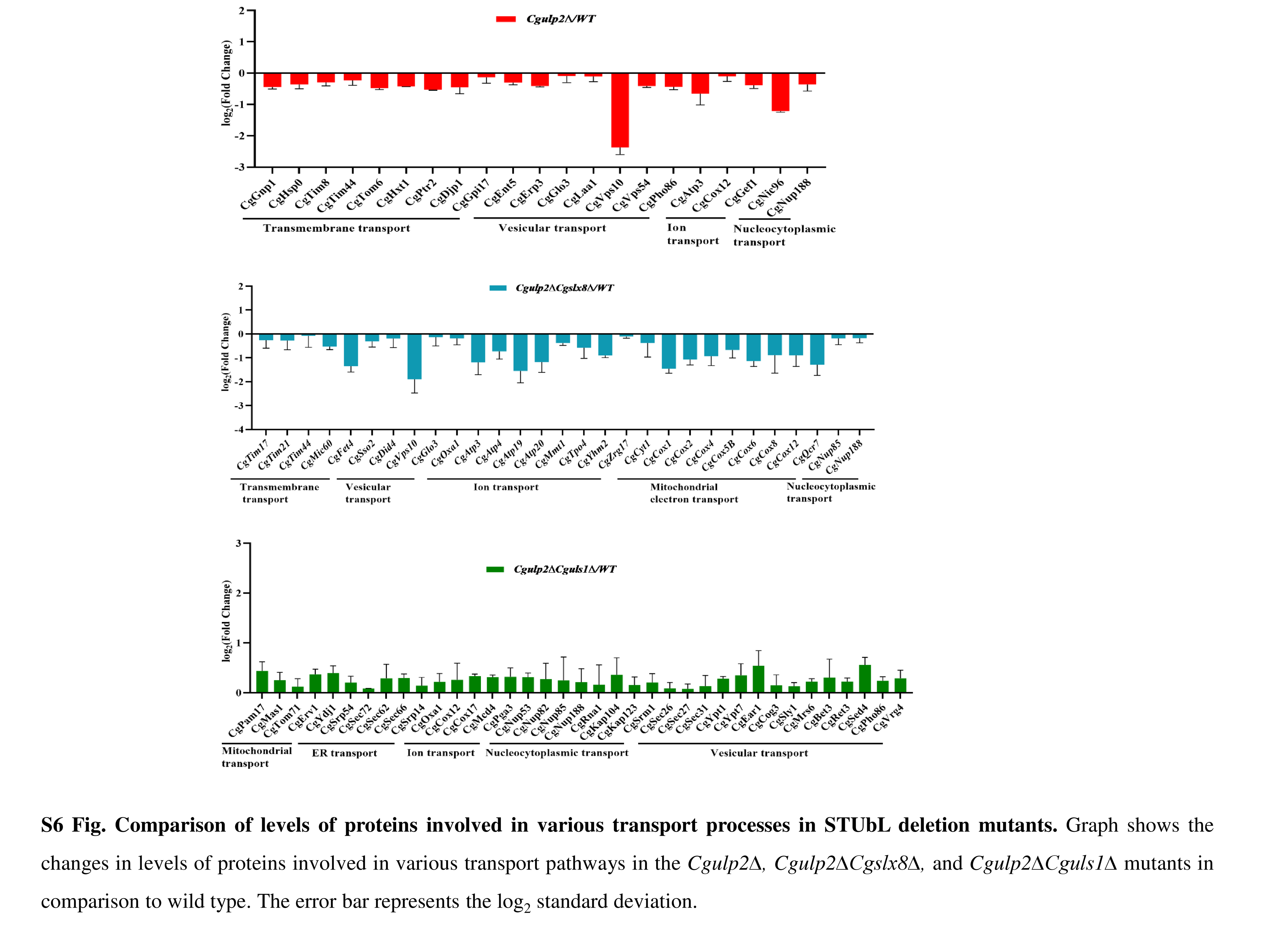

Supplement: S6 Fig — Graph shows the change in levels of proteins involved in various transport pathways in the Cgulp2Δ, Cgulp2ΔCgslx8Δ, and Cgulp2ΔCguls1Δ mutants in comparison to wild type. The error bar represents the log2 standard deviation. (TIF) [file ppat.1012742.s006.tif]

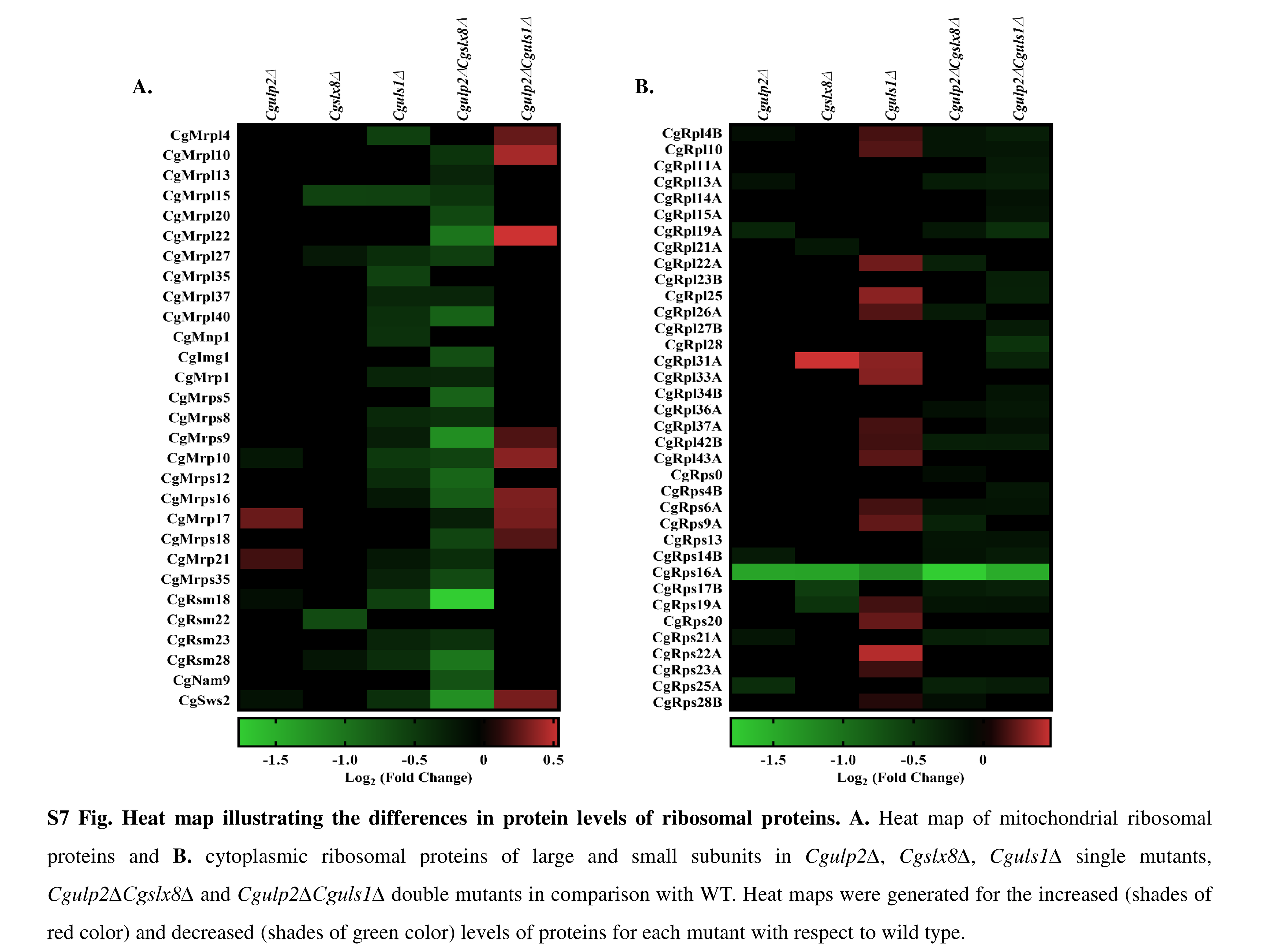

Supplement: S7 Fig — A. Heat map of mitochondrial ribosomal proteins and B. cytoplasmic ribosomal proteins of large and small subunits in Cgulp2Δ, Cgslx8Δ, Cguls1Δ single mutants, Cgulp2ΔCgslx8Δ and Cgulp2ΔCguls1Δ double mutants in comparison with WT. Heat maps were generated for the increased (shades of red color) and decreased (shades of green color) levels of proteins for each mutant with respect to wild type. (TIF) [file ppat.1012742.s007.tif]

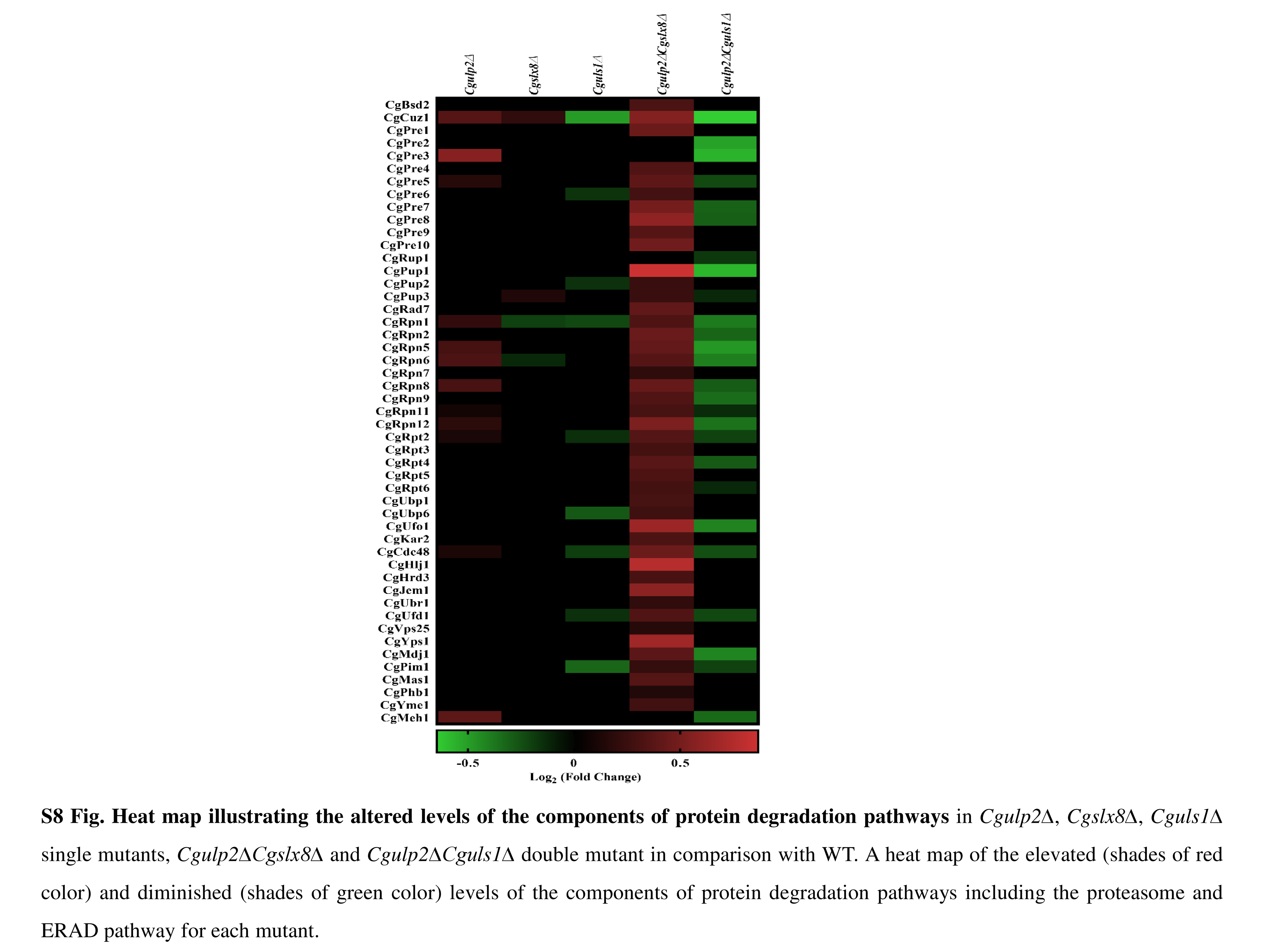

Supplement: S8 Fig — A heat map of the elevated (shades of red color) and diminished (shades of green color) levels of the components of protein degradation pathways including the proteasome and ERAD pathway for each mutant. (TIF) [file ppat.1012742.s008.tif]

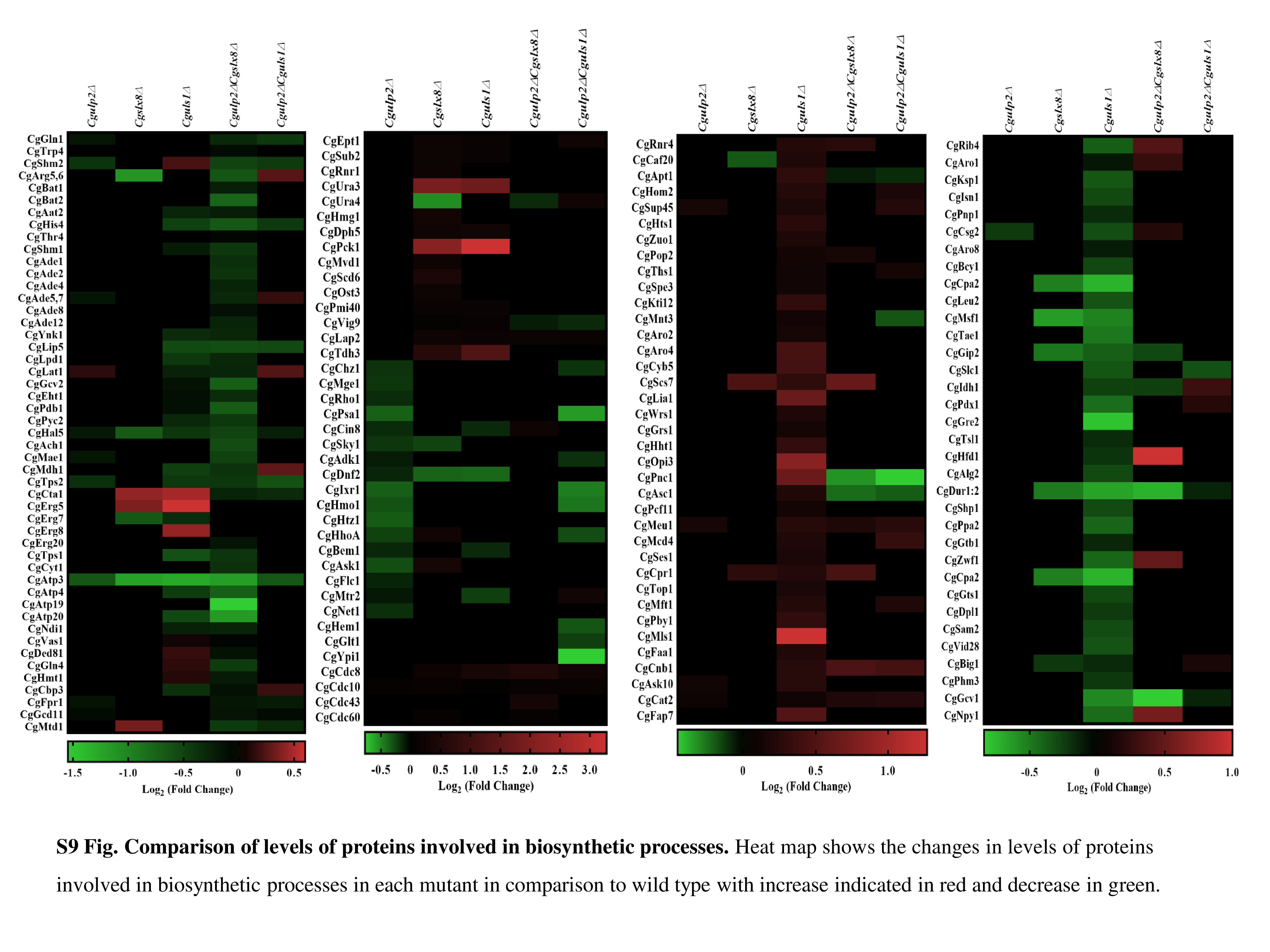

Supplement: S9 Fig — Heat map shows the changes in levels of proteins involved in biosynthetic processes in each mutant in comparison to wild type with increase indicated in red and decrease in green. (TIF) [file ppat.1012742.s009.tif]

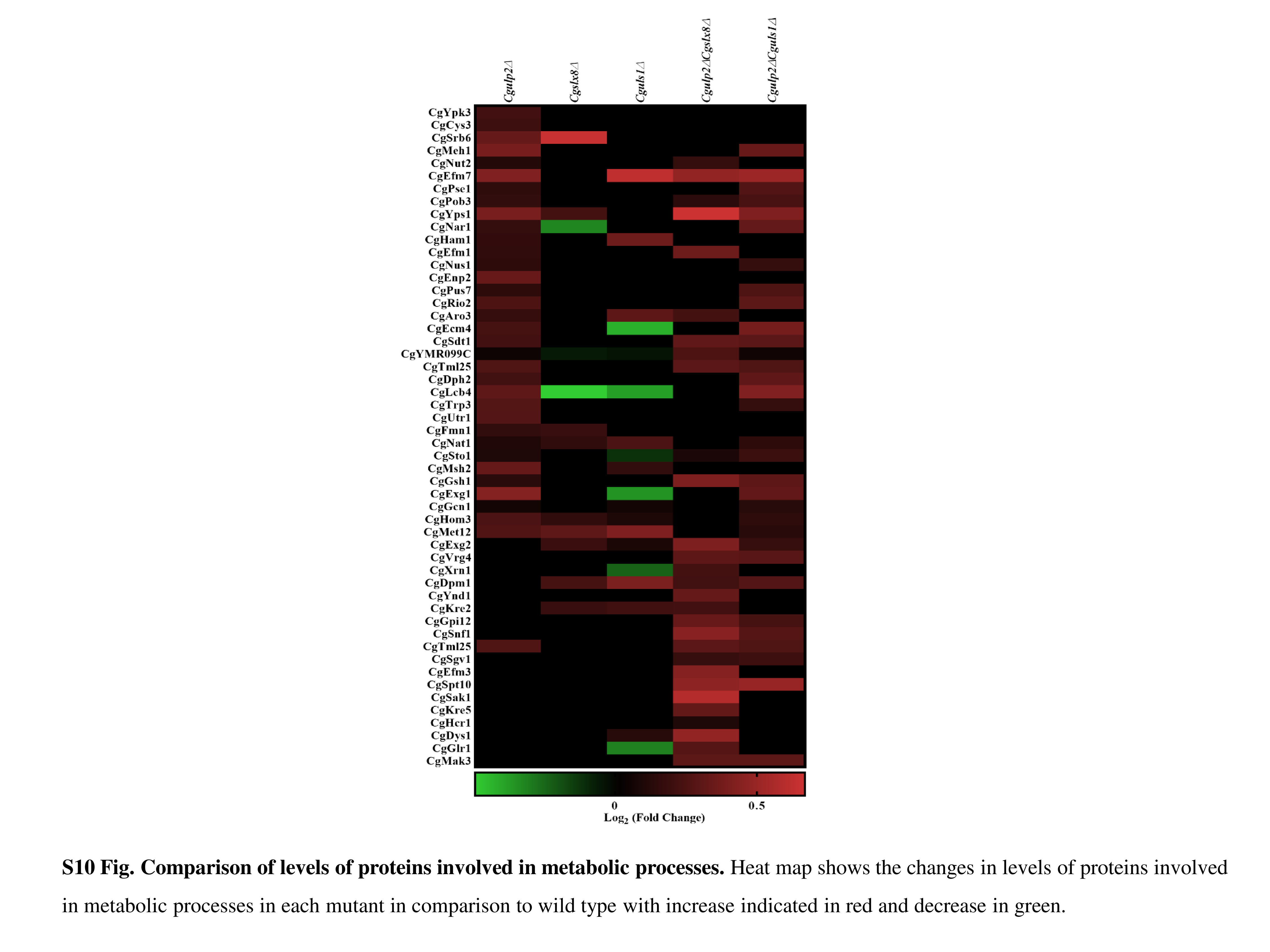

Supplement: S10 Fig — Heat map shows the changes in levels of proteins involved in metabolic processes in each mutant in comparison to wild type with increase indicated in red and decrease in green. (TIF) [file ppat.1012742.s010.tif]

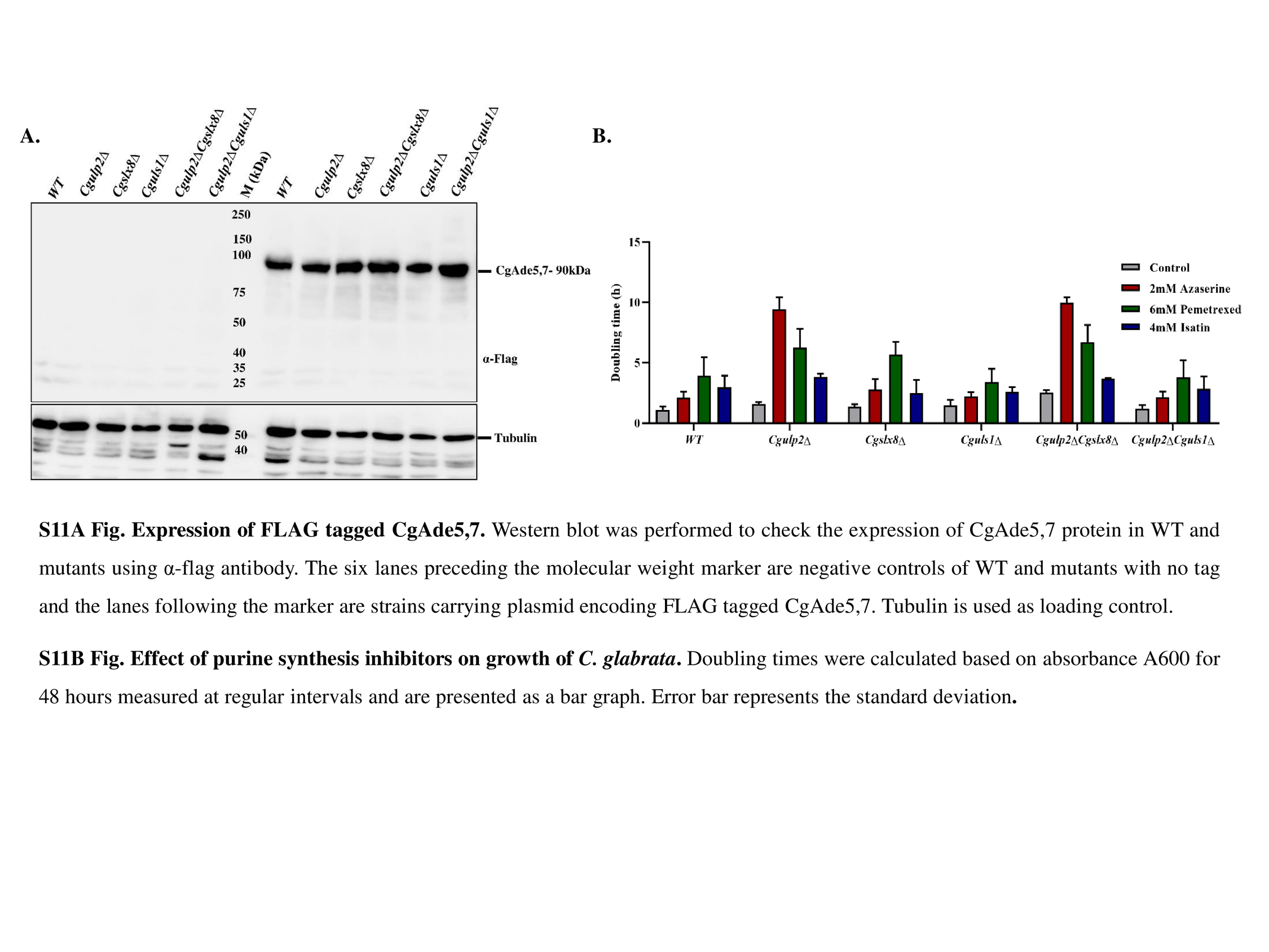

Supplement: S11 Fig — A. Expression of FLAG tagged CgAde5,7. Western blot was performed to check the expression of CgAde5,7 protein in WT and mutants using α-flag antibody. The six lanes preceding the molecular weight marker are negative controls of WT and mutants with no tag and the lanes following the marker are strains carrying plasmid encoding FLAG tagged CgAde5,7. Tubulin is used as loading control. B. Effect of purine synthesis inhibitors on growth of C. glabrata. Doubling times were calculated based on absorbance A600 for 48 hours measured at regular intervals and are presented as a bar graph. Error bar represents the standard deviation. (TIF) [file ppat.1012742.s011.tif]

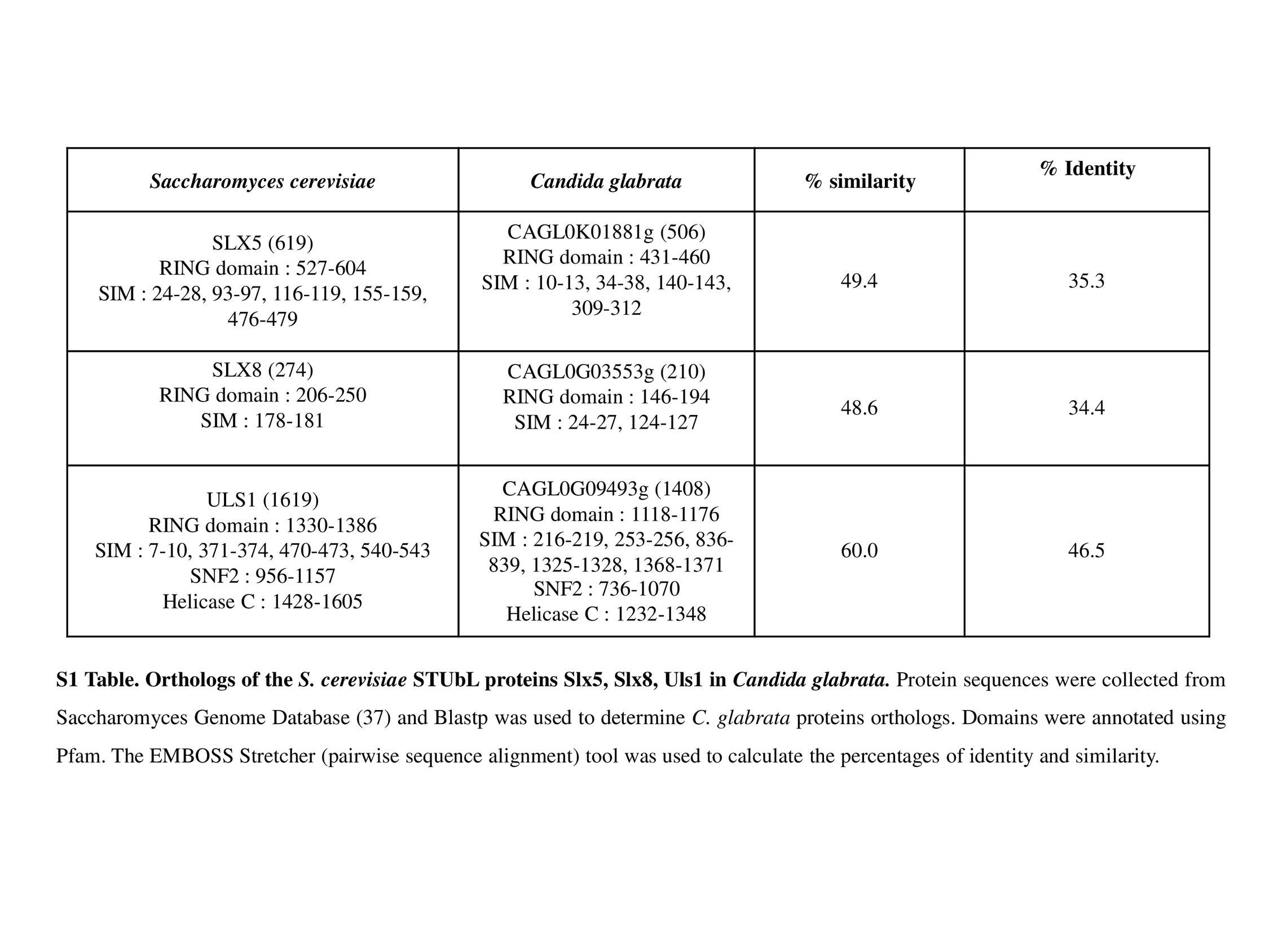

Supplement: S1 Table — Protein sequences were collected from Saccharomyces Genome Database [37] and Blastp was used to determine C. glabrata orthologs. Domains were annotated using Pfam. The EMBOSS Stretcher (pairwise sequence alignment) tool was used to calculate the percentages of identity and similarity. (TIF) [file ppat.1012742.s012.tif]
