# Supplementary material for: SUMO-targeted Ubiquitin Ligases as crucial mediators of protein homeostasis in Candida glabrata
Source: PLoS Pathog. 2024 Dec 6;20(12):e1012742. doi: 10.1371/journal.ppat.1012742 (PMC11654969; doi:10.1371/journal.ppat.1012742)
Supplement: Attachment — Submitted filename: response to comments.docx [file ppat.1012742.s023.docx]

Date: 29/10/2024

Dear Dr. Morschhäuser ,

Thank you very much for reviewing our manuscript and your comments. We have extensively revised the manuscript to add new text, correct the existing text, performed a new experiment and made new figures to improve the quality of the presentation as suggested by the reviewers.

I hope you will now find the manuscript suitable for publication in PLoS Pathogens.

We thank the reviewers for their thorough review and insightful suggestions which have improved the focus of the manuscript.

Please find below our point by point response to the comments made by the reviewers

Krishnaveni Mishra

**Comment:**

Two reviewers also suggest to test the virulence of the mutants in animal infection models, since the paper claims that the STUbLs regulate pathogenicity. I would not be surprised if the disturbance of a central cellular function reduces the fitness of a pathogen in its host and do not insist on such experiments. If you do not wish to add animal infection experiments, however, the title of the paper and corresponding statements in the text should be modified accordingly.

Response: Thank you for your comments. Yes, we agree that the mutants are likely to reduce the infectivity and survival in the animal as well. In addition, we have already shown that loss of *ulp2* leads reduced colonization and survival in mouse models.

We have modified the title to remove “pathogenesis” and also in other places in the text.

A question that occurred to me when reading the paper: Do you have an explanation for why a proteasome inhibitor (MGI32) does so strongly (ca. 10-fold increase in CFUs) improve the growth of C. glabrata wild-type cells even in the absence of stress conditions (Fig. 6E)?

Response: Thank you for this comment, we have also been puzzled by this observation. And have searched literature to see if there is any example of this, but found none. We reasoned that the protein degradation pathways maybe more active in *C. glabrata* in general and addition of MG132 improves growth by preserving some of the proteins that are at low levels. The strong link we observe between overall SUMO proteome-proteasome is not seen in S. cerevisiae. We have now mentioned this in the discussion.

We cannot make any decision about publication until we have seen the revised manuscript and your response to the reviewers' comments. Your revised manuscript is also likely to be sent to reviewers for further evaluation.

Responses to Reviewer Questions

**Part I - Summary**
Please use this section to discuss strengths/weaknesses of study, novelty/significance, general execution and scholarship.

Reviewer #1: In this study, the authors investigated the roles of SUMOylation in Candida glabrata. They constructed deletion mutants of various genes involved in this pathway and conducted phenotypic characterizations. They found that Ulp2, a deSUMOylase, is essential for maintaining protein homeostasis, and that the loss of both Ulp1 and the ubiquitin ligase Slx8 leads to heightened protein degradation, making the cells susceptible to various stressors, such as high temperature and DNA-damaging agents. Importantly, ulp2, slx6, and ulp2 slx8 mutants fail to proliferate in macrophages. Proteomic analyses revealed that these mutations significantly compromise purine biosynthesis and mitochondrial functions.

This is the first comprehensive study of the role of SUMOylation in C. glabrata. It identified genes and cellular processes crucial for stress tolerance and survival in macrophages, suggesting potential targets for antifungal therapies.

While the data suggest that genes such as ULP2 and SLX8 may also be important for pathogenicity, the study does not include data on virulence using animal infection models.

Response: Thank you. We have earlier shown that loss of Ulp2 affects the ability of *C. glabrata* to colonize organs in mice. We have reasoned that these mutants which again affect the same pathway are also likely to have similar effects and have refrained from doing animal experiments

To enhance the readability of the paper, the authors should improve their use of proper punctuation, which is missing in many places.

Response: We have edited the manuscript for grammar and punctuation and believe this revised version has enhanced readability.

Reviewer #2: The manuscript by Gupta et al presents findings that characterise the roles of the deSUMOylase Ulp1 and the SUMO-targeting ubiquitin ligases Slx5, Slx8 and Uls1 in Candida glabrata. Characterisation of a set of single and double mutants in genes encoding these proteins is presented (e.g. growth under various conditions, stress responses, growth within immune phagocytes), alongside biochemical studies and proteome analyses to understand the growth phenotypes of the mutants. The study presents a relatively detailed analyses of the phenotypes of the mutants in vitro, and the biochemistry supports the conclusion that these proteins, as expected, are required for protein homeostasis. As a consequence, inactivation of these pathways leads to pleiotropic phenotypes. Mitochondrial morphology and function, as well as purine biosynthesis seems to be particularly affected. At the level of mechanism, the data is consistent with Ulp1 and not Slx5/Slx8 being the main SUMO-targeting ubiquitin ligase for proteins that are de-ubiquitinated by Ulp1. In contrast enhanced degradation of Ulp1 targets through the proteasome pathways is seen in the ulp1 slx8 mutant, potentially explaining the various phenotypes.

Reviewer #3: In this report, Gupta et al. follow up on their analysis of the C. glabrata desumoylase Ulp2, and show interesting genetic interactions between the disturbance of protein sumoylation caused by deletion of ULP2, and the function of the SUMO-dependent ubiquitin ligases (STUbL) Uls1 and Slx5/Slx8. In particular, they find that the ulp2 slow growth phenotype can be suppressed by further deletion of ULS1 (but not of SLX8), under several conditions (although under other conditions, such as hydroxyurea, the phenotype is exacerbated). They go on to show that the level of SUMOylated proteins, which is reduced in ulp2, is recovered in the ulp2 uls1 mutant, leading to the suggestion that the phenotype of the ulp2 deSUMOylase mutant is due to increased protein degradation vie the STUbL Uls1 and the proteasome. This model is then corroborated by showing that addition of the proteasome inhibitor MG132 can rescue both SUMOylated protein levels and growth in the ulp2 mutant. The authors then carried out mass spec analysis of the different single and double mutants and focus on a few pathways expected, based on the MS data, to be affected: purine biosynthesis and mitochondrial function. Additional observations are that all the mutants analyzed become sensitive to killing by macrophages and are defective in biofilm formation.

**Comment**: This paper contains many interesting observations, but I found it to be a very difficult read. One problem is that the authors spend a lot of time framing their work as addressing the antifungal drug crisis in general, and in the Candida clade (they emphasize C. auris) in particular. In reality however, C. glabrata is phylogenetically much closer to S. cerevisiae than to all other Candida spp., so that generalization of any result from C. glabrata to other Candida spp. is problematic. And while one might argue that C. glabrata is an important pathogen in its own right and devising therapies against it is a worthwhile goal, the notion that a universally conserved pathway (the human host also contains deSUMOylases and STUbLs) represents a useful drug target is not convincing.

**Response:** We have presented the work in the context of potential pathways that could be antifungal targets and that Ulp2 in particular seems to be important for virulence and /or pathogenesis in fungi where it has been studied.

We have reduced the text in the first two paragraphs that talks about antifungals in the Introduction and also in the discussion section to reduce the emphasis of this aspect. The introduction and discussion do elaborate on the SUMOylation/ deSUMOylation/proteostasis axis.

**Comment:** A second problem is that while the paper contains a coherent and interesting core, namely the observations described in Figs. 2, 4, 5 and 6, as well as the MS analysis, I find the follow up on the MS analysis (Figs. 8 and 9), disconnected from the main story and, in the case of purine biosynthesis, unconvincing (see detailed comments below). I would recommend an extensive re-write that would put the focus on the molecular biology aspects of the SUMO/STUbL/proteasome axis.

**Response:**

We have expanded the results and discussion section to emphasize the SUMO-proteasome axis. The purine biosynthesis and the mitochondrial defects observed have just been described in the results and we have not emphasized them in the discussion. The emphasis in the discussion is on the SUMO-STUbL-Proteasome axis

**Part II – Major Issues: Key Experiments Required for Acceptance**
Please use this section to detail the key new experiments or modifications of existing experiments that should be absolutely required to validate study conclusions.

Generally, there should be no more than 3 such required experiments or major modifications for a "Major Revision" recommendation. If more than 3 experiments are necessary to validate the study conclusions, then you are encouraged to recommend "Reject".

Reviewer #1:

**Commment:**  (1) In the ulp2 slx8 mutant, mitochondrial complexes II, III, IV, and V were significantly downregulated. Since mutations affecting the mitochondrial ETC have been linked to antifungal resistance in various Candida species, including C. glabrata (e.g., 10.1111/mmi.15229), the authors should also perform antifungal susceptibility tests on the ulp2 and slx8 ulp2 mutants, given their resistance to various stresses.

**Response**: We performed antifungal sensitivity tests for the mutants . We find that Δulp2, *Δulp2slx8* are generally more sensitive (new figure 1E) than wild type and the *Δulp2uls1* is more resistant or similar to wt. This could be due to either their compromised mitochondrial function as reported in 10.1111/mmi.15229 or due to altered protein homeostasis of the targets of these antifungals. We have included this data and discussed it in the text.

Comment:
(2) Despite the demonstrated importance of ULP2 and SLX8 in stress tolerance and survival macrophages, this study provides limited insights into the pathogenicity of C. glabrata. It would be helpful to provide some data on virulence using animal infection models.

Response: In our previous work we had performed mice experiments for Ulp2 deletion and established that the colonization was reduced significantly. We therefore believe that loss of components that further exacerbate these phenotypes or alter SUMO proteome so profoundly are likely to have strong effects on colonization of organs in animals.

Reviewer #2: My comments and suggestions are below.
• I might have missed this, but from a mechanism point of view, since Slx8 is a ubiquitin ligase it is unclear why protein degradation would be enhanced in its absence. It seems as if the suggestion is that upregulation of Uls1 is the possible reason. However, this is only seen in the ulp2 slx8 mutant, and therefore might be indirect. Further discussion on this point would be beneficial.

**Response:** We agree with the reviewer that this is counterintuitive that loss of *slx8* leads to reduced accumulation of SUMOylated proteins and indeed is different from what one observes in *S. cerevisiae*. In fact, the triple mutant of slx5slx8uls1 in S. cerevisae is viable and still shows higher molecular weight SUMO conjugates (PMID: 17728242). Taken together with our inability to generate slx8uls1 double nulls and the different phenotypes observed for *slx5* in comparison with *S. cerevisiae*, we think the regulation of these STUbLs is different from *S. cerevisiae* that warrants further study.

Importantly, this suggests that the SUMO-STUbL-Proteasome axis is differently controlled and perhaps stronger in proteostasis in *C. glabrata* than in *S. cerevisiae.* We have discussed this now in the revised manuscript.

The Uls1 being a major STUbL is also derived from the observation that loss of uls1 in both slx5 and ulp2 mutants leads to reappearance of higher molecular weight conjugates.

**Comment**
• Given the relatedness of C. glabrata and S. cerevisiae, and the high conservation of the pathways under study across organisms, I would suggest that it would be beneficial to present a more detailed discussion on the conserved vs diverged roles of Ulp2, Slx5 /Slx8 and Uls1 in C. glabrata compared to what we know from other organisms in terms of mechanism, mutant phenotypes, genetic interactions, essentiality of genes or not etc.

Response: We have expanded the discussion in the revised manuscript to provide brief details of the phenotypes associated with the STUbLs and Ulp2 in other organisms and also highlighted the differences we have observed between *S. cerevisiae* and *C. glabrata.*

• Changes in mitochondrial morphology presented in Figure 9 are not obvious. I would suggest more detailed microscopy images of representative cells and quantification of the changes.

**Response:** The figures have been replaced with a different panel with more clear morphology. The morphology has also been quantified and added to Figure 8B, C for clarity in the revised manuscript.

• Please explain in figure legends the nature of the replicates (biological or technical) and define what the error bars represent. At present, this information is available for some panels, but not all (across all figures).

Response: We apologize for the oversight. Now all legends and figures contain this information

Reviewer #3: 1) Fig. 1 and Table 1 – in silico protein comparison – is not very informative. I suggest displaying it as supplementary information, if at all.

**Response**: We have moved the figure to the sup material as for comparison of the proteins being studied here (Ulp2, Slx5,8 and Uls1) between *S. cerevisiae* and *C. glabrata* we feel the figure might be useful. To provide context for the origin of this work, we have retained a brief description in the results section.

**comment**
2) Fig. 4 and p. 13: here and throughout the paper, the authors refer to the SUMO-reactive bands on the Western blot as “poly-SUMOylation”. However it is not clear to me why these bands couldn’t equally represent mono-SUMOylated proteins.

**Response:** Thank you, the interpretation is correct. We have replaced polySUMO with higher molecular weight SUMO conjugates as suitable.

**Comment**:
3) pp. 21-23 and Fig. 8: the authors found by MS that anabolic proteins, and in particular purine biosynthesis, are affected in the STUbL mutants. The protein ADE5,7 seems the most affected. However, is only reduced 10-20% in ulp2 and ulp2 slx8, and is up 10% in ulp2 uls1. To demonstrate that these difference are physiologically significant, the authors then proceed to show that overexpression of ADE5,7 can suppress the slow growth of ulp2 and ulp2 slx8, and that the STUbL mutants are more sensitive to purine pathway inhibitors. However,

1) while these mutant strains indeed exhibit better growth with the ADE5,7 overexpression plasmid, so does at 42° also the ulp2 uls1 strain, which has a higher level of the protein to begin with (Fig. 8C). In fact, according to Figs 8D, E, so do all the strains, including the wild type, regardless of ADE5,7 protein levels, suggesting this could be a plasmid effect.

**Response:** We do observe improved growth in all strains, but the improvement at 30^o^C is very small in all strains except the *Δslx8Δulp2*. A plasmid effect should have similar levels of improvement in all strains. Since the effect is significantly stronger in some strains, we think that it is the elevated expression of Ade5,7 that supports better growth of the *Δslx8Δulp2*.

At 42^o^C, all strains that are sensitive to growth at this temperature show improved growth when Ade5,7 is expressed. It is possible that the levels of Ade5,7 are not same at 30^o^C and 42^o^C and hence Δ*ulp2uls1*, which grows poorly at 42^o^C also shows improved growth. The mass spectrometry was done at 30^o^C and therefore the levels of Ade5,7 at 42 ^o^C are not known. In *S. cerevisiae*, ade5,7 strains are reported to be “innately thermosensitive”; reduced ade5,7 may make *Candida glabrata* strains temperature sensitive

I also note that the ADE5/7 OE strains and their controls are not spelled out in either the legend or the Methods section.

**Response:** The details have been added to the legend of the new figure 7.

2) In Fig. 8F-I, the authors show inhibition of growth by purine pathway inhibitors, and they suggest that the higher inhibition detected for some of the strains correlates with lower gene expression. However, it is hard to gauge, from the figures shown, which differences are significant.

**Response**: In the revised manuscript, significance values have been added to the figure

Furthermore, contrary to expectation, no resistance is detected in the naturally ADE5,7 overexpressing strain *Δulp2 uls1*. Thus, I do not find that the authors have convincingly show physiological relevance of the MS data regarding the purine biosynthesis pathway.

**Response:** The Δuls1Δulp2 strain is as resistant as the wild type in most cases. Since none of the inhibitors directly inhibit the Ade5,7 enzyme, we cannot expect one-one correlation. The sensitivity is because overall purine biosynthesis is lowered in the strains and any further reduction by inhibiting other enzymes in the pathway will lower the already low purine levels. We reasoned that azaserine, which inhibits the enzyme that produces the substrate for Ade5,7 has maximum effect because now the substrate is not available even for the lowered levels of Ade5,7. The inhibitors downstream of this pathway require more inhibitor to show any effect because the residual activity in the presence of the inhibitor is sufficient to carry on purine biosynthesis with the available product of Ade5,7 activity.

4) Looking at the proteasome proteins just in ulp2 slx8 (Fig. S7) vs the Excel sheet S4, I see that Pup1 is most induced in the Excel, but appears black in Fig. S7; while the bright red Hlj1 is only moderately induced according to the Excel. I recommend to re-check all S6-S9 figures vs. the data in the Excel sheet.

**Response**: Thank you very much for this important detail that was misrepresented in our original manuscript. Heat map has been corrected in the revised manuscript and all data in excel to heat map has been rechecked.

The error arose because there are two Pup1 in Cg database. One (CAGL0LO4312g) is equivalent to ScPup1 which is upregulated in Δulp2Δslx8 double and is a proteasomal component. The second Pup1, CAGL0M12947g, is a Pdr1 up-regulated gene, which is mitochondrial (PMID21408004) and mistakenly shown in the heat map. This has been corrected now.

**Part III – Minor Issues: Editorial and Data Presentation Modifications**
Please use this section for editorial suggestions as well as relatively minor modifications of existing data that would enhance clarity.

Reviewer #1: Line 116 at least
Lines 118-119. To understand the importance of STUbLs in C. glabrata,,

**Response:** Edited in revised manuscript

**Comment**
Ulp2 was described as SUMO specific isopeptidases, deSUMOylase, deSUMOylating enzyme, or SUMO protease in different places, which could be confusing for readers who are not familiar with topic.

**Response:** Ulp2 is now described as deSUMOylase and SUMO protease in the revised manuscript.

Line 143 and throughout the manuscript, the prefix 'Cg' can be removed from all gene and protein names, as readers are unlikely to confuse them with those from other species. The prefix should be used only when two or more species are being compared or described together. This will make the text easier to read and the figures less crowded.

Response: We have not removed the prefix because in searches, when these gene names appear, unless one reads the paper carefully, one may mistake it for S. cerevisiae protein. We note that in most papers of *C. glabrata* this convention is followed for distinguishing the Cg and Sc proteins. Also both in introduction and discussion we do talk about the Sc protein equivalents and if we keep shifting between the two nomenclatures for the Cg proteins, it might be difficult for the readers to comprehend.

Figure 2 The number of strains shown in the heat map in Figure 2D exceed that used in panels A-C. The ‘+’ symbol used in the colour scale does not make sense and should be removed together with the ‘–’ symbol.

Response : Figure 2 is now new figure 1. Figure 1A presents the growth curve of all generated strains in YPD medium, displayed in two separate graphs. Figure 1B illustrates the growth of selected strains in RPMI medium. In figure 1C, the phenotypes of all the strains are now shown, and figure 1D has been modified accordingly by removing the colour scale

Line 239. The description is not clear. After 8 hours of what? No matter what, it is not reflected in Figure 3A and B.

Response: In the revised manuscript, the description has been clarified in legends. It now specifies that after 8 hours of adding *C. glabrata* to macrophages, the yeast cells began to divide and increase in number. This observation was captured in the WT infections during the experiment (figure 2A and B now), reflecting the replication of *C. glabrata* cells.

Figure 3A-B. I suggest changing ‘Macrophage internalized cells’ to ‘Survival in macrophage’.

**Response:** It changed to "survival in macrophage” in revised manuscript

Line 261. Replace the asterisk symbol with X. Also, change ‘C. glabrata strains to ‘C. glabrata cells’.

**Response:** This has been replaced and edited in revised manuscript

Line 266. in macrophage-internalized

Line 284. at least

Line 297. Ponceau S

Response: All three lines have been edited in the revised manuscript

Line 447. The citation format here is different from the rest.

**Response:** The citation style has been reviewed, and all references have been updated to follow the Vancouver format consistently throughout the manuscript.

Figure 8A. The bars of different colours, red, green and blue, are not explained.

**Response:** It has now been explained in the legend of the revised manuscript.

Lines 498-499. How the cells were grown was not described. In liquid medium? how long was the growth period?

**Response:** The manuscript has been revised to include detailed information on how the cells were grown, including the use of liquid medium and the duration of the growth

Figure 8F-H. The Y-axis represents the percentage of inhibition. What does the height of the grey bars indicate? Typically, the growth of the vehicle control should be set to one, and the growth of the drug-treated cells should be expressed as a percentage relative to the vehicle control.

**Response:** The height of the grey bars represented the vehicle control (now removed). The bar graphs have been modified accordingly, with the vehicle control growth set to 100%, and the Y-axis now indicates % growth.

Line 513. mitochondrial not moitochondrial.

Response: Corrected

Lines 513-516, This long sentence needs proper punctuation marks. Line 514, in the Cgulp2ΔCgslx8Δ double mutant as complexes II, III, IV, and V.

Response: The sentence is split and punctuation added in the revised manuscript

Line 612. a few
Line 653. homologues
Line 668. uncover new targets for antifungal therapies.
Line 669. Replace ‘a lot of’ with ‘multiple’, and ‘at once’ with ‘simultaneously, ’.
Lines 671-672. …homeostasis, offering an additional approach for designing combinatorial therapies.

Response: edited line 612, 653, 668, 669, 671-672 in revised manuscript

Lines 672-673. Please provide the references for the statement ‘For instance, some of the purine biosynthesis inhibitors are already used as antifungals in the clinic’.

Response: In the revised manuscript, reference has been added

Line 676. design of

Response: Corrected in the revised manuscript

Reviewer #2: (No Response)

Reviewer #3: 1) Slx5 and Slx8 are expected to function as a heterodimer , and indeed this is how the authors analyze them. However the phenotype of the individual slx5 and slx8 mutants are not always coherent, most notably at 42° and at the level of SUMOylation (Fig. 4A). This merits discussion.

Response: Thank you for pointing out this omission. We have now included this and other differences in the revised manuscript in the discussion section

2) I couldn’t help noticing, while scanning the MS data, that Smt3/SUMO itself is significantly reduced (about 35%) in all mutants except uls1. I wonder how this observation can be fitted in the author’s model of the SUMO/STUbL/proteasome axis.

Response: We reason that this is perhaps due to increased degradation due to lack of recycling of SUMO in these mutants compared to wild type.

In normal wild type cells, SUMO conjugates are removed by Ulp2 (and Ulp1) and the SUMO is recycled generating a pool of free SUMO. In the absence of CgUlp2, deSUMOylation activity is impaired, causing an accumulation of polySUMOylated proteins that are ubiquitinated by STUbLs, leading to their degradation by the proteasome. Since, the proteasome does not distinguish between substrate and modifier, when polySUMOylated proteins are degraded, the SUMO moieties are also degraded. This results in depletion of available SUMO pools since SUMO is not effectively recycled. Normally, Ulp2 cleaves SUMO from substrates to facilitate its reuse, but without Ulp2, this recycling is disrupted, and polySUMO chains are more likely to be degraded rather than disassembled. This increased targeting of polySUMOylated proteins for proteasomal degradation reduces free Smt3/SUMO levels.

Additionally, the stress on the SUMO/proteasome axis due to Ulp2 loss may overwhelm the cell's feedback mechanisms, potentially downregulating SUMO synthesis or impairing the maturation of SUMO precursors, further reducing SUMO availability.

3) The observation in Fig. S7 and Fig. 7 that the proteasome proteins are over-represented in ulp2 uls1 and under-represented in ulp2 slx8 correlates nicely with the absence and presence of sumoylated bands in the Westerns of these strains, and with the MG132 effect. I didn’t see this observation discussed in the text.
Response: Thank you for the suggestion. We have expanded the discussion on this observation in the revised manuscript by discussing the correlation between proteasome protein levels and sumoylation patterns in the Western blot analysis, as well as the effects of MG132 treatment in the results. This is now included in the section on the proteasomal degradation component heat map and further elaborated in the discussion as well in the revised manuscript.

4) The color scheme for “up” and “down” proteins is opposite in Fig. 7 vs. Fig. S6-S9. This is confusing.

**Response:** We have revised the color scheme in the newly designated Figure 6 to align with the heat map, ensuring consistency with Figures S7-S10.

5) For Fig. 7, the significance of the X axes is not clear. Does "% protein" mean average protein levels or number of proteins in the category that are up or down? If the latter, then putting the percentages in the negative range of the axis doesn't make sense.

**Response:** The "% protein" represents the proportion of proteins that fall into specific categories of either upregulated or downregulated proteins. This has now been modified in the updated Figure 6, where both upregulated and downregulated proteins are displayed in the same direction in the revised manuscript.

6) Since the paper deals only with C. glabrata, the Cg prefix is not really required and removing it from all gene and protein names might make the text more readable.
Response: We have not removed the prefix because in searches, when these gene names appear, unless one reads the paper, one may mistake it for S. cerevisiae protein. We note that in most papers of *C. glabrata* this convention is followed for distinguishing the Cg and Sc proteins. Also both in introduction and discussion we do talk about the Sc protein equivalents and if we keep shifting between the two nomenclatures for the Cg proteins, it might be difficult for the readers to comprehend
Typos

l. 24: opportunistic
l. 71: should be “the epsilon amino group of lysine in target proteins”
l. 81: isopeptidases
l. 116: at least
l. 169: should be “The Cgulp2ΔCgslx8Δ strain was …”
l. 513 moitochondrial
l. 567 Molecualr

Response: All these and other typos have been corrected in the revised manuscript.

throughout: “thermal sensitivity” should be “temperature sensitivity”

Response: Replaced thermal sensitivity with temperature sensitivity in the revised manuscript
